# Serum Osteopontin Predicts Degree of Hepatic Fibrosis and Serves as a Biomarker in Patients with Hepatitis C Virus Infection

**DOI:** 10.1371/journal.pone.0118744

**Published:** 2015-03-11

**Authors:** Yasuhiro Matsue, Mikihiro Tsutsumi, Nobuhiko Hayashi, Takashi Saito, Mutsumi Tsuchishima, Nobuyuki Toshikuni, Tomiyasu Arisawa, Joseph George

**Affiliations:** 1 Department of Hepatology, Kanazawa Medical University, Uchinada, Ishikawa, Japan; 2 Department of Gastroenterology, Kanazawa Medical University, Uchinada, Ishikawa, Japan; SAINT LOUIS UNIVERSITY, UNITED STATES

## Abstract

**Background & Aims:**

Osteopontin (OPN) is a matricellular protein that upregulates during pathogenesis of hepatic fibrosis. The present study was aimed to evaluate whether serum OPN could be used as a biomarker to assess the degree of hepatic fibrosis in patients with hepatitis C virus (HCV) infection.

**Methods:**

Needle biopsy was performed on HCV patients and scored as zero fibrosis (F0), mild fibrosis (F1), moderate fibrosis (F2), severe fibrosis (F3) and liver cirrhosis (F4) based on Masson’s trichrome and α-smooth muscle actin (α-SMA) staining. Serum OPN levels were measured using ELISA and correlated with the degree of fibrosis. Furthermore, the OPN values were correlated and evaluated with platelets count, serum hyaluronic acid (HA), and collagen type IV and subjected to receiver operating characteristic (ROC) curve analysis.

**Results:**

Serum OPN levels were remarkably increased from F0 through F4 in a progressive manner and the differences were significant (P < 0.001) between each group. The data were highly correlated with the degree of hepatic fibrosis. The ROC curve analysis depicted that serum OPN is an independent risk factor and an excellent biomarker and a prognostic index in HCV patients.

**Conclusions:**

The results of the present study indicate that serum OPN levels reflect the degree of hepatic fibrosis and could be used as a biomarker to assess the stage of fibrosis in HCV patients which would help to reduce the number of liver biopsies. Furthermore, serum OPN serves as a prognostic index towards the progression of hepatic fibrosis to cirrhosis and hepatocellular carcinoma.

## Introduction

Hepatic fibrosis is characterized by excessive synthesis and deposition of connective tissue components especially fibrillar collagens in the extracellular matrix of the liver [1–3]. Fibrogenesis is the result of continuous response to a chronic liver injury such as alcohol, viral hepatitis, drugs, toxins, nonalcoholic steatohepatitis (NASH), cholestasis, and metabolic disorders [4, 5]. In the fibrogenesis *milieu*, there is upregulation of several molecules and proteins, synthesis and release of numerous cytokines and growth factors and a perpetual injury and healing of the liver tissue that lead to fibrosis and cirrhosis [6, 7]. Uncontrolled hepatic fibrosis leads to the distortion of cellular architecture leading to nodular formation, liver cirrhosis, and HCC and ultimate death [8].

Osteopontin (OPN) is an extracellular matrix protein and was first cloned and sequenced in 1986 [9]. It is a multifunctional matricellular protein that plays a significant role in innate immunity, cell survival, tumor invasion, and angiogenesis [10, 11]. OPN is expressed in a variety of cells including fibroblasts, macrophages, dendritic cells, endothelial cells and smooth muscle cells [12]. During hepatic fibrogenesis, OPN promotes activation of quiescent hepatic stellate cells and increases collagen I expression and secretion [13]. Furthermore, OPN involves in the pathogenesis of hepatocellular carcinoma (HCC) [14]. Recently, it was reported that OPN level correlates with degree of fibrosis in patients with alcoholic liver disease (ALD) [15].

Hepatitis C virus (HCV) is a major cause of liver disease worldwide and a potential cause of substantial morbidity and mortality [16]. It is estimated that approximately 170 million people worldwide are chronically infected with HCV [17]. The persistent infection of HCV will lead to the development of fibrosis and cirrhosis due to perpetual and impaired wound-healing process [18, 19]. This repeated repair and regeneration process could results in genomic aberrations and mutations which lead to the development of HCC [20, 21]. Almost all HCV patients have various stages of fibrosis in their liver tissue which is classified as mild fibrosis (F1), moderate fibrosis (F2), severe fibrosis (F3), and liver cirrhosis (F4) [22]

The incidence of development of HCC increases parallel with the progression of hepatic fibrosis in patients with HCV infection [23]. Therefore, it is important to assess and evaluate the degree of hepatic fibrosis in HCV infected patients. Even though liver biopsy has its gold standard for the accurate evaluation of the degree of fibrosis, liver biopsy is not feasible in all patients due to various reasons. Recently it has been reported that OPN play a significant role in alcoholic hepatitis both in human and experimental animals [24].The aim of the current investigation was to evaluate the use of serum OPN as a useful biomarker to assess the degree of liver fibrosis in patients with HCV infection.

## Materials and Methods

### Subjects

All patients involved in the study were admitted to the Kanazawa Medical University hospital for diagnosis and treatment from April 2012 through October 2014. The patients group, age, and sex are provided in Table 1. The study protocol was reviewed and approved by the Ethical and Clinical Investigation Committee of Kanazawa Medical University, Japan and the investigation has been conducted according to the principles expressed in the Declaration of Helsinki (revised 2013). Written consent was obtained from each patient prior to the procedure after full explanation of the purpose of the study. All patients were examined by ultrasound (US) and/or computed tomography (CT) scan to detect HCC and diagnose liver cirrhosis (LC). Patients diagnosed with HCC were not included in the study. All the selected patients were tested positive for HCV RNA in the blood by PCR analysis and also to HCV antibodies in the sera, as determined with an EIA third-generation testing kit (Ortho-Clinical Diagnostics, Tokyo, Japan). The patients had not received antiviral or immunomodulating therapy or not had a history of alcoholism. The patients were also screened for hepatitis B virus (HBV) markers such as HBsAg, HBsAb, and HBcAb in their sera and found to be negative.

**Table 1:** Basic and clinical parameters of F0, F1, F2, F3, and F4 groups of patients selected for the study (N = 115).

Patients Group	Age (Mean ± SD)	Sex (M/F)	AST (IU/liter) (Mean ± SD)	ALT (IU/liter) (Mean ± SD)	γ-GT (IU/liter) (Mean ± SD)	Albumin (g/100 mL) (Mean ± SD)
F0	65 ± 6.3	5/6 (N = 11)	23.2 ± 5.0	25.6 ± 7.5	28.5 ± 8.3	4.5 ± 0.3
F1	62 ± 7.7	12/16 (N = 36)	35.3 ± 10.8	36.4 ± 14.5	25.7 ± 11.7	4.2 ± 0.3
F2	58 ± 5.9	17/9 (N = 26)	56.6 ± 17.1*	76.5 ± 34.9***	61.5 ± 34.5**	4.2 ± 0.3
F3	63 ± 3.6	11/11 (N = 22)	43.9 ± 14.1*	48.4 ± 22.9*	29.8 ± 9.3	3.9 ± 0.2*
F4	69 ± 4.8	10/10 (N = 20)	54.2 ± 5.8**	48.2 ± 15.5*	38.0 ± 9.7	3.4 ± 0.4***

*P < 0.05

**P < 0.01, and

***P < 0.001 compared to F0

### Liver biopsy

Percutaneous liver biopsy using disposable needle (18 g x 16 cm, 22 mm penetration depth), (Bard Max-Core, Tempe, AZ) was performed on all selected patients in each group. The biopsy specimens were fixed in 10% phosphate buffered formalin for a minimum period of 24 hrs and processed in an automatic tissue processor optimized for liver, embedded in paraffin blocks, and cut into sections of 4 μm thickness. The paraffin sections were deparaffinized and stained with hematoxylin & eosin (H&E) following conventional protocol. Masson’s trichrome staining for connective tissue was carried out employing a kit (#K037, Poly Scientific, Bay Shore, NY). The stained sections were examined with an optical microscope (Olympus BX50, Tokyo, Japan) and photographed using DP71 digital camera (Olympus, Tokyo, Japan) attached to the microscope.

### Criteria for classification of the degree of liver fibrosis

In the current study we included only patients with proven cases HCV infection. After evaluation of H&E and Masson’s trichrome staining of the liver biopsy specimens by an experienced hepatopathologist, the patients were classified according to the method of Desmet *et al*. [22] for the classification of chronic hepatitis. The degree of fibrosis was classified as F0 = no fibrosis; F1 = portal fibrosis (many thin collagen fibers surrounding portal tracts); F2 = moderate fibrosis (many thick fibers connecting periportal to midzonal areas or portal-portal septa); F3 = bridging fibrosis (many septa connecting portal triad and central veins); F4 = cirrhosis (nodular cirrhosis with extensive network of collagen fibers).

### Immunohistochemical staining for α-smooth muscle actin

The immunohistochemical staining for α-smooth muscle actin (α-SMA) in biopsy liver sections was carried out using a broad-spectrum histostain kit (Cat# 85-9943, Invitrogen, Carlsbad, CA). In brief, the paraffin sections were deparaffinized and treated with 3% hydrogen peroxide (H_2_O_2_) for 5 min followed with blocking serum for 30 min. The sections were rinsed in PBS and treated with α-SMA antibody (Cat#, M0851, Dako Corporation, Carpinteria) and incubated overnight in a moisturized chamber at 4°C. The slides were then washed in PBS three times and treated with biotinylated second antibody followed with streptavidin peroxidase. The final stain was developed using the substrate 3-amino-9-ethylcarbazole (AEC) which produce a deep red color on antigen sites. The slides were counterstained with Mayer’s hematoxylin and mounted using an aqueous-based mounting medium (Dako, Carpinteria, CA) and photographed.

### Measurement of AST, ALT, γ-GT, albumin, type IV collagen and HA in serum

Blood samples were collected from all patients involved in the study and serum was separated by conventional methods. Aspartate transaminase (AST), alanine transaminase (ALT), γ-glutamyl transpeptidase (γ-GT), and albumin in the serum were measured using an auto-analyzer. AST, ALT, γ-GT values are presented as International Units per liter (IU/liter) and albumin as g/100 mL of serum. Blood platelets were counted using an auto-analyzer.

Collagen type IV present in the serum samples was determined using radioimmunoassay which recognizes 7S domain in type IV collagen [25]. The assay kit (#KCLD1) was procured from Mitsubishi Chemical Medience, Tokyo, Japan. Assay was performed according to the manufacturer’s instructions and the results are presented as ng/mL serum. Hyaluronic acid (HA) concentrations in serum were determined using ELISA-based sandwich HA-binding assay kit (Chugai Diagnostics, Tokyo, Japan), which follow the method of Chichibu *et al*. [26]. Serum HA levels are presented as ng/mL.

### Measurement of osteopontin in serum

Osteopontin concentrations present in the serum of HCV infected patients were measured using human OPN ELISA kit (Cat#: ab100618, Abcam, Chuo-ku, Tokyo, Japan) as per the manufacturer’s instructions. In brief, the serum samples were diluted 20 fold with the assay diluent provided in the kit. A 20 fold dilution of serum is necessary, especially for the samples from patients with LC in order to obtain an absorbance value within the range of standards used. Recombinant human OPN (provided in the kit) ranging from 0–18 ng/ml was used as standards. Exactly, 100 μl of diluted serum sample or standard was added to a microplate pre-coated with anti-human OPN antibody, sealed the microplate with the special cover provided in the kit, and incubated overnight at 4°C with gentle shaking. The solution was discarded, washed the wells four times with the wash buffer (provided in the kit), and blotted off the plate. Then added 100 μl of biotinylated second antibody and incubated for 1 hr at room temp with gentle shaking. The solution was discarded, washed the plate again four times with wash buffer, blotted off the plate, added 100 μl of diluted HRP conjugated-streptavidin, and incubated for 45 min at room temp with shaking. The microplate was again washed for 5 times, added 100 μl of tetramethylbenzidine (TMB), and incubated exactly for 30 min in the dark with shaking. Finally, 50 μl of stop solution (2 M sulfuric acid) was added and the intensity of the color was measured immediately on a microplate reader at 450 nm. A standard curve was prepared on Microsoft Excel software in log-log mode and plotted a linear graph. The amount of OPN present in the serum samples was calculated from the regression equation obtained from the curve.

### Statistical analysis

Arithmetic mean and standard deviation were calculated for all quantitative data and presented as Mean ± SD. The results were statistically evaluated using one-way analysis of variance (ANOVA). The F0 mean values were compared with F1, F2, F3, and F4 mean values using least significant difference method. The value of *P* < 0.05 was considered as statistically significant. Pearson and Lee’s correlation coefficient was used to evaluate the correlation between the staining intensity of collagen and α-SMA with serum OPN levels in HCV infected patients. Receiver operating characteristic (ROC) curve analysis was used to evaluate the diagnostic accuracy of serum OPN, platelet count, HA, and collagen type IV for discriminating fibrosis stages. During the study period, approximately two-thirds of the patients were used for a derivation cohort and the remaining patients were used for a validation cohort. Moreover, the whole cohort was also used for another validation cohort. Optimal cut-off values were determined on the basis of Youden index [27]. Delong et al. [28] method was used to compare the area under the ROC curves (AUCs) and multiple comparisons were corrected using Bonferroni test [29].

## Results

### Basic and clinical parameters of patients selected for the study

The basic and clinical parameters of patients selected for the study are presented in Table 1. There were fluctuations in AST, ALT, and γ-GT levels between F1 to F4 groups compared to F0 and some of the values were significantly different. But serum albumin levels were decreased from F1 to F4 groups in a sequential manner and the mean albumin values in F3 and F4 groups were significantly reduced (*P* < 0.05 and *P* < 0.001, respectively) compared to F0 group.

### Masson’s trichrome and α-smooth muscle actin staining in liver biopsy sections

**Fig 1:**
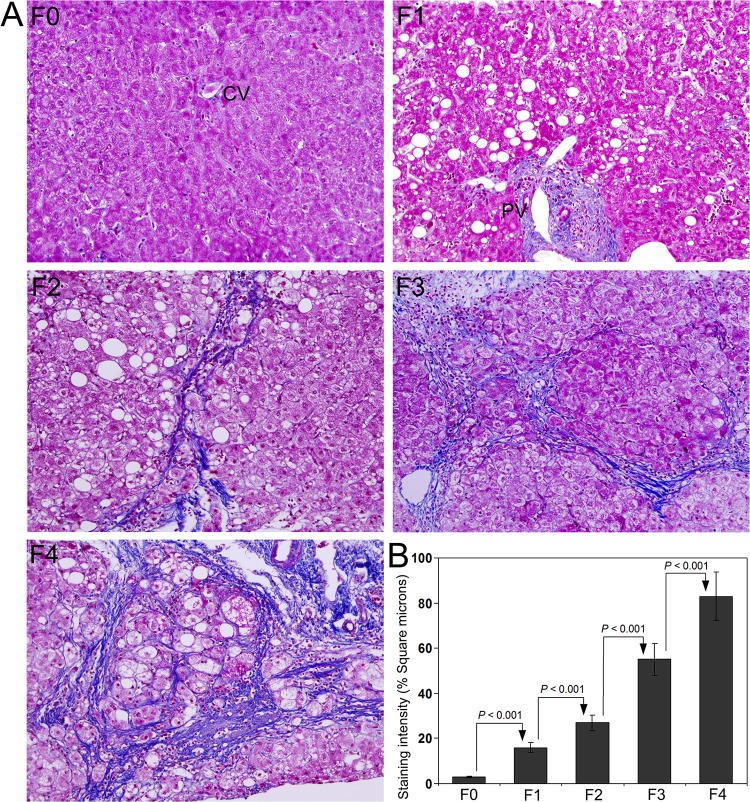
Masson’s trichrome staining for collagen in liver biopsy specimens from patients with HCV infection. **(A)** Representative staining image from the group of patients classified as F0, F1, F2, F3, and F4 depends on the degree of fibrosis. **(B)** Quantification of Masson’s trichrome staining using Image-Pro discovery software. The data are mean ± S.D. in each group. Original magnification, x100.

Masson’s trichrome staining to demonstrate the deposition of mature collagen fibers in the representative liver biopsy specimens from stage F0 through F4 is presented in Fig. 1A. As per the classification of chronic hepatitis, fibrosis was completely absent in F0. Many thin collagen fibers were present surrounding portal tracts in F1. Samples with septal fibrosis were classified as F2. Distinct and clear bridging fibrosis was present in stage F3. Marked nodular cirrhosis was present in all samples included in F4. Steatosis was present in almost all cases from F1 through F4. Quantification of the intensity of Masson’s trichrome staining demonstrated a significant increase from F0 through F4 and the difference between each group was highly significant (*P* < 0.001) (Fig. 1B).

**Fig 2:**
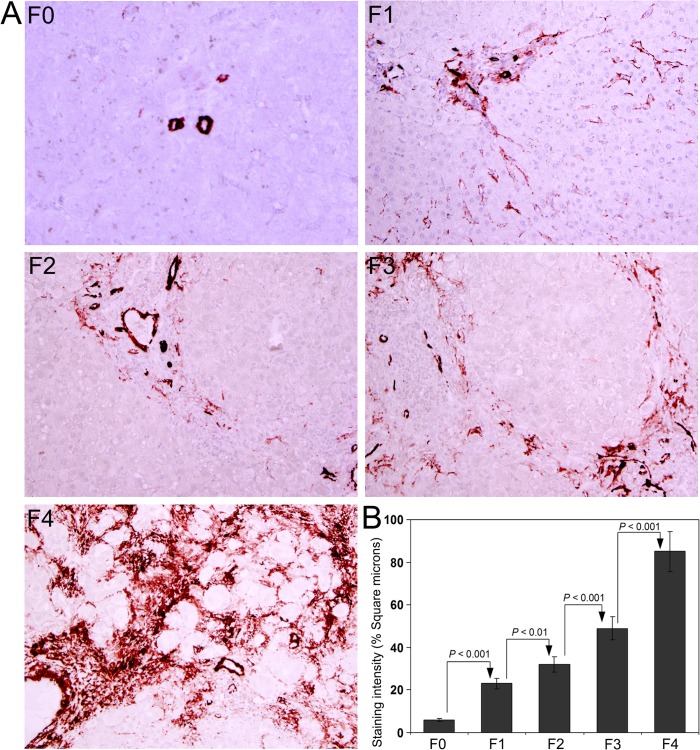
Immunohistochemical staining for α-smooth muscle actin (α-SMA) in the liver biopsy sections. **(A)** The intensity of α-SMA staining in activated stellate cells coincided with the degree of fibrosis in F0, F1, F2, F3, and F4 group of patients with HCV infection. **(B)** Quantification of α-SMA staining. The data are mean ± S.D. in each group. Original magnification, x100.

Immunohistochemical staining for α-SMA to demonstrate the activation of hepatic stellate cells is presented in Fig. 2A. Since activated hepatic cells are mainly responsible for the synthesis of collagen during fibrogenesis, the intensity of α-SMA staining will be positively correlated with the deposition of collagen and degree of fibrosis. Quantitative analysis of the staining intensity of α–SMA is depicted in Fig. 2B. A sequential increase was present in the staining intensity from F0 through F4 and the difference between each group was significant at 0.1% level (*P* < 0.001), except between F1 and F2, where the difference was significant at 1% level (*P* < 0.01).

### Blood platelets, serum type IV collagen and HA in patients with HCV infection

**Fig 3:**
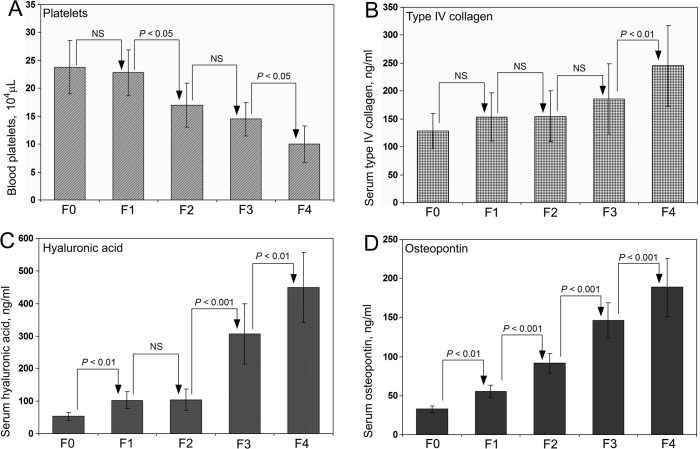
Parameters to assess the degree hepatic fibrosis. Blood platelets count **(A)**, serum type IV collagen **(B)**, serum hyaluronic acid **(C)**, and serum OPN **(D)** levels in F0, F1, F2, F3, and F4 group of patients with HCV infection. Among the 4 biomarkers depicted to assess the stage of hepatic fibrosis, only serum OPN demonstrated a sequential increase from F0 through F4 with a significant difference (*P* < 0.001) between each group that coincides with the degree of fibrosis.

Platelets count in the blood, type IV collagen and HA levels in the serum of patients with HCV infection are presented in Fig. 3A, 3B, & 3C, respectively. None of these three parameters demonstrated a sequential increase or decrease with a significant difference between each groups. There was a marked increase in the case of serum HA in groups F3 and F4 compared to F0, but the difference was not significant between F1 and F2. It is well known that serum HA will increase dramatically in all sorts of fibrosis but not an appropriate parameter to distinguish between different stages of any type of fibrosis.

### Serum OPN levels in patients with HCV infection

Serum OPN concentrations in HCV infected patients classified according to the degree of fibrosis are presented in Fig. 3D. A sequential increase was observed in serum OPN level from F0 through F4. The difference was significant between all groups at 0.1% level (*P* < 0.001) except between F0 & F1, where the difference was significant at 1% level (*P* < 0.01). The increase and the statistical difference between the groups are coincided with the degree of fibrosis evaluated through Masson’s trichrome and α–SMA staining. The data demonstrated that serum OPN is an ideal biomarker to predict the degree of hepatic fibrosis in HCV infected patients without carrying out a liver biopsy.

### Association of serum OPN and degree of hepatic fibrosis

Pearson and Lee’s correlation coefficient analysis was used to assess the linear regression between the staining intensity of collagen and α-SMA with serum OPN concentrations in HCV infected patients. Correlation analysis between the mean staining intensity of collagen and the mean serum OPN concentration in different grades of fibrosis (F0 through F4) revealed a highly significant positive correlation (r = 0.994). Similarly there was a significant positive correlation (r = 0.985) between the mean staining intensity of α-SMA and mean serum OPN concentration indicating the relationship between the activation of liver stellate cells and expression of OPN in the hepatic tissue during fibrogenesis.

### Competence of serum OPN to predict the degree of hepatic fibrosis

**Fig 4:**
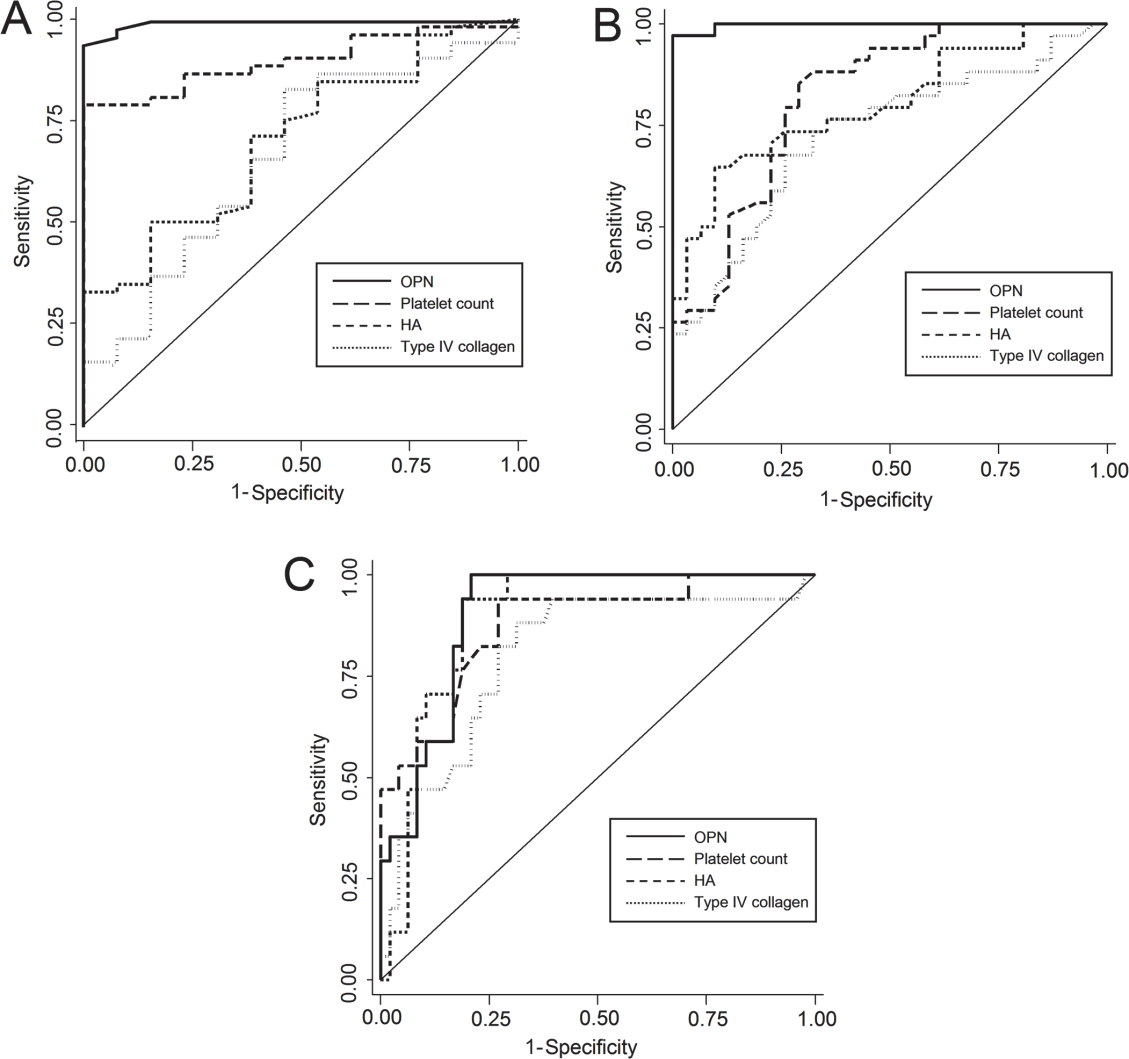
Receiver Operating Characteristic (ROC) curve analysis to evaluate the competence of serum OPN as a diagnostic marker for the degree of hepatic fibrosis in patients with HCV infection. (**A**) ROC curves of serum OPN, platelet count, HA, and collagen type IV for discriminating F1 against F2/F3/F4. (**B**) ROC curves of serum OPN, platelet count, HA, and collagen type IV for discriminating F1/F2 against F3/F4. (**C**) ROC curves of serum OPN, platelet count, HA, and collagen type IV for discriminating F1/F2/F3 against F4. OPN, osteopontin; HA, hyaluronic acid; F, fibrosis.

In order to evaluate the competence of serum OPN to discriminate the degree of hepatic fibrosis, we compared serum OPN levels with blood platelets count, serum HA, and type IV collagen. In addition, to increase reliability of serum OPN as a biomarker for estimating the stage of fibrosis, the whole cohort was divided into a derivation and a validation cohort. The total number of patients in the derivation cohort was 65 (F1 = 15, M/F = 7/8, Mean age 66 ± 9.6; F2 = 16, M/F = 9/7, Mean age 57 ± 12.9; F3 = 17, M/F = 9/8, Mean age 64 ± 8.8; F4 = 17, M/F = 8/9, Mean age 70 ± 8.4). In the derivation cohort group, discrimination analysis of F1 from F2/F3/F4, the AUC value of serum OPN was 0.996 (cut-off value, 83 ng/mL; sensitivity, 94.2%; specificity, 100%); which was the highest and significantly different from the other serum markers (Table 2 and Fig. 4A). Similarly, for the discrimination of F1/F2 from F3/F4, the AUC of serum OPN was 0.997 (cut-off value, 124 ng/mL; sensitivity, 97.1%; specificity, 100%); the value was also highest and significantly different from other parameters (Table 3 and Fig. 4B). In the discrimination analysis of F4 from F1/F2/F3, the AUC of serum OPN was 0.904 (cut-off value; 152 ng/mL; sensitivity, 100%; specificity, 79.2%) and the value was not significantly different from other parameters (Table 4 and Fig. 4C).

**Table 2:** Comparison of AUCs for discriminating F1 against F2/F3/F4 in the derivation cohort (N = 65).

Parameters	Cut-off	Sensitivity (%)	Specificity (%)	PPV (%)	NPV (%)	AUC (95% CI)	*P*-value after Bonferroni correction*
Osteopontin	83 ng/mL	94.2	100	100	81.3	0.996 (0.945–1.000)	
Platelet count	169x10^6^/mL	78.9	100	100	54.2	0.898 (0.791–0.956)	0.048
Hyaluronic acid	138 ng/mL	50.0	84.6	92.9	29.7	0.704 (0.582–0.814)	0.0007
Collagen type IV	131 ng/mL	83.7	53.9	87.8	43.8	0.661 (0.534–0.774)	0.0006

*The AUC of serum OPN was compared with the AUCs of other serum markers.

F, fibrosis; PPV, positive predictive value; NPV, negative predictive value; AUC, area under the receiver operating characteristic curve; CI, confidence interval

**Table 3:** Comparison of AUCs for discriminating F1/F2 against F3/F4 in the derivation cohort (N = 65).

Parameters	Cut-off	Sensitivity (%)	Specificity (%)	PPV (%)	NPV (%)	AUC (95% CI)	*P*-value after Bonferroni correction*
Osteopontin	124 ng/mL	97.1	100	100	96.9	0.997 (0.945–1.000)	
Platelet count	156x10^6^/mL	85.3	71.0	76.3	81.5	0.822 (0.700–0.901)	0.0032
Hyaluronic acid	179 ng/mL	64.7	90.3	88.0	70.0	0.803 (0.682–0.889)	0.0014
Collagen type IV	165 ng/mL	67.7	74.2	74.2	67.6	0.728 (0.598–0.827)	0.0001

*The AUC of serum OPN was compared with the AUCs of other serum markers.

F, fibrosis; PPV, positive predictive value; NPV, negative predictive value; AUC, area under the receiver operating characteristic curve; CI, confidence interval

**Table 4:** Comparison of AUCs for discriminating F1/F2/F3 against F4 in the derivation cohort (N = 65).

Parameters	Cut-off	Sensitivity (%)	Specificity (%)	PPV (%)	NPV (%)	AUC (95% CI)	*P*-value after Bonferroni correction*
Osteopontin	152 ng/mL	100	79.2	63.0	100	0.904 (0.810–0.965)	
Platelet count	135x10^6^/mL	94.1	72.9	55.2	97.2	0.876 (0.772–0.945)	1.000
Hyaluronic acid	179 ng/mL	94.1	81.3	64.0	97.5	0.895 (0.791–0.956)	1.000
Collagen type IV	166 ng/mL	88.2	68.8	50.0	94.3	0.805 (0.682–0.889)	0.576

*The AUC of serum OPN was compared with the AUCs of other serum markers.

F, fibrosis; PPV, positive predictive value; NPV, negative predictive value; AUC, area under the receiver operating characteristic curve; CI, confidence interval

The AUCs for discriminating hepatic fibrosis stage in the validation cohort and the whole cohort were calculated for OPN and compared with the AUCs of other serum markers and the results are presented as Table 5 and Table 6, respectively. The total number of patients in the validation cohort was 39 (F1 = 21, M/F = 5/16, Mean age 58 ± 8.8; F2 = 10, M/F = 8/2, Mean age 59 ± 9.7; F3 = 5, M/F = 2/3, Mean age 61 ± 7.6; F4 = 3, M/F = 2/1, Mean age 61 ± 10.4). In the validation cohort group, the *P*-value after Bonferroni correction was < 0.05 when the AUC of serum OPN discriminating F1 vs F2/F3/F4 compared to the AUC of platelet count, serum HA, and collagen type IV discriminating F1 vs F2/F3/F4 and also when the AUC of serum OPN discriminating F1/F2 vs F3/F4 compared to the AUC of platelet count and serum HA discriminating F1/F2 vs F3/F4 (Table 5). In the whole cohort, the *P*-value after Bonferroni correction was 0.0004 or less when the AUC of serum OPN discriminating F1 vs F2/F3/F4 compared to the AUC of platelet count, serum HA, and collagen type IV discriminating F1 vs F2/F3/F4. Similarly, the *P*-value after Bonferroni correction was < 0.0001 when the AUC of serum OPN discriminating F1/F2 vs F3/F4 compared to the AUC of platelet count, serum HA, and collagen type IV discriminating F1/F2 vs F3/F4 (Table 6). Thus, both in the derivation and validation cohorts, the discriminant power of serum OPN was higher than that of conventional serum markers.

**Table 5:** Comparison of AUCs for discriminating hepatic fibrosis stage in the validation cohort (N = 39).

Parameters	Discrimination	Cut-off	Sensitivity (%)	Specificity (%)	PPV (%)	NPV (%)	AUC (95% CI)	*P*-value after Bonferroni correction
	F1 vs F2/3/4	83 ng/mL	83.3	100	100	87.5	1.000 (0.910–1.000)	
Osteopontin	F1/2 vs F3/4	124 ng/mL	100	100	100	100	1.000 (0.910–1.000)	
	F1/2/3 vs F4	152 ng/mL	100	91.6	50.0	100	1.000 (0.910–1.000)	
	F1 vs F2/3/4	169×10^6^/mL	66.7	81.0	75.0	73.9	0.738 (0.579–0.870)	0.007*
Platelet count	F1/2 vs F3/4	156×10^6^/mL	50.0	71.2	33.3	85.2	0.714 (0.551–0.850)	0.006^#^
	F1/2/3 vs F4	135×10^6^/mL	33.3	77.8	11.1	93.3	0.782 (0.635–0.907)	0.083^@^
	F1 vs F2/3/4	138 ng/mL	44.4	95.2	88.9	66.7	0.747 (0.579–0.870)	0.006*
Hyaluronic acid	F1/2 vs F3/4	179 ng/mL	37.5	87.1	42.9	84.4	0.675 (0.498–0.809)	0.036^#^
	F1/2/3 vs F4	179 ng/mL	66.7	86.1	28.6	96.9	0.667 (0.498–0.809)	0.653^@^
	F1 vs F2/3/4	131 ng/mL	88.9	57.1	64.0	85.7	0.820 (0.665–0.925)	0.042*
Collagen type IV	F1/2 vs F3/4	165 ng/mL	62.5	80.6	45.5	89.3	0.897 (0.758–0.971)	0.121_#_
	F1/2/3 vs F4	166 ng/mL	33.3	72.2	9.1	92.9	0.815 (0.665–0.925)	0.219^@^

The AUC of serum OPN was compared with the AUCs of other serum markers.

F, fibrosis; PPV, positive predictive value; NPV, negative predictive value; AUC, area under the receiver operating characteristic curve; CI, confidence interval

(*, F1 vs F2/F3/F4

^#^, F1/F2 vs F3/F4

^@^, F1/F2/F3 vs F4)

**Table 6:** Comparison of AUCs for discriminating hepatic fibrosis stage in the whole cohort (N = 104).

Parameters	Discrimination	Cut-off	Sensitivity (%)	Specificity (%)	PPV (%)	NPV (%)	AUC (95% CI)	*P*-value after Bonferroni correction
	F1 vs F2/3/4	83 ng/mL	90.0	100	100	82.9	0.997 (0.965–1.000)	
Osteopontin	F1/2 vs F3/4	124 ng/mL	95.2	100	100	96.9	0.999 (0.965–1.000)	
	F1/2/3 vs F4	152 ng/mL	100	84.5	60.6	100	0.945 (0.879–0.979)	
	F1 vs F2/3/4	169×10^6^/mL	75.7	88.2	93.0	63.8	0.827 (0.740–0.894)	0.0004*
Platelet count	F1/2 vs F3/4	156×10^6^/mL	78.6	72.6	66.0	83.3	0.802 (0.708–0.870)	< 0.0001^#^
	F1/2/3 vs F4	135×10^6^/mL	85.0	75.0	44.7	95.5	0.861 (0.784–0.924)	0.288^@^
	F1 vs F2/3/4	138 ng/mL	48.6	91.2	91.9	46.3	0.763 (0.666–0.838)	< 0.0001*
Hyaluronic acid	F1/2 vs F3/4	179 ng/mL	59.5	88.7	78.1	76.4	0.794 (0.708–0.870)	< 0.0001^#^
	F1/2/3 vs F4	179 ng/mL	90.0	83.3	56.3	97.2	0.886 (0.807–0.939)	0.623^@^
	F1 vs F2/3/4	131 ng/mL	84.3	55.9	79.7	63.3	0.747 (0.656–0.830)	< 0.0001*
Collagen type IV	F1/2 vs F3/4	165 ng/mL	66.7	77.4	66.7	77.4	0.783 (0.687–0.854)	< 0.0001^#^
	F1/2/3 vs F4	166 ng/mL	80.0	70.2	39.0	93.7	0.822 (0.730–0.886)	0.113^@^

The AUC of serum OPN was compared with the AUCs of other serum markers.

F, fibrosis; PPV, positive predictive value; NPV, negative predictive value; AUC, area under the receiver operating characteristic curve; CI, confidence interval

(*, F1 vs F2/F3/F4

^#^, F1/F2 vs F3/F4

^@^, F1/F2/F3 vs F4)

## Discussion

Chronic infection of HCV is one of the most dangerous diseases affecting worldwide today, especially in many Asian countries. Generally, the infection with HCV is asymptomatic until the patients develop extensive liver fibrosis or cirrhosis. Liver biopsy is a cumbersome procedure and in most cases patients do not agree for a biopsy unless they have serious complications. So it is very important to explore an appropriate diagnostic and prognostic serum biomarker to evaluate the grade of fibrosis in patients with HCV infection. We found that serum OPN concentrations are significantly increased in all patients with HCV infection and the levels are highly correlated with the degree fibrosis assessed using the staining intensity of α-SMA and total collagen in liver biopsy specimens. Measurement of serum OPN concentration would be definitely a useful parameter to evaluate the degree of fibrosis, especially in asymptomatic patients with HCV infection. Depends on the serum OPN concentration, doctors could decide whether patients need to undergo a liver biopsy for further evaluation.

Osteopontin is involved in various physiological process and pathological conditions including inflammation, cirrhosis, angiogenesis, and progression of almost all sorts of cancers [30]. Although the function of OPN is not completely understood, it has been demonstrated that OPN participates in the recruitment of macrophages during inflammation, acts as a survival or mitogenic factor for epithelial and vascular cells during cancer progression, and is closely associated with extracellular matrix synthesis and pathogenesis of fibrosis [31]. Since serum OPN levels are elevated in various pathological disorders, it is important to have primary diagnosis of the disease to identify the cause of increased serum OPN levels. OPN is a highly labile protein and the serum samples should be assayed for OPN immediately after collection or the samples should be stored at -80°C for up to 15 days without freeze/thawing cycles. Our repeated attempts to stain OPN protein employing different antibodies (ab8448: Abcam; AB1870: Millipore; 10011: IBL-Japan) in paraffin embedded tissue sections prepared from the liver biopsy samples that are fixed in phosphate-buffered formalin were not successful. The most probable reason for this could be the destruction of OPN antigen during tissue processing.

This is the first study to compare serum OPN levels with conventional serum markers to assess the degree of liver fibrosis in HCV infected patients. We found that serum OPN concentration is significantly correlates with the degree of fibrosis and could be used as a biomarker towards the course of disease progression. The results demonstrated that serum OPN distinguished F1, F2, and F3 more sharply than blood platelet count, serum HA, and collagen type IV, even though all the four serum markers studied differentiated F4 stage from the other fibrosis stages. Recently, many blood tests have been proposed as alternatives to liver biopsy for identifying the degree of fibrosis or cirrhosis [32]. Among these, aspartate aminotransferase–platelet ratio index (APRI) and Fibro-Test have been validated for patients with HCV infection [32]. In the current study also we calculated APRI, but found that the discriminative ability of APRI was poor compared to serum OPN. Among all available blood tests and parameters to assess the grade of fibrosis in patients with chronic HCV infection, we concluded that serum OPN concentration is the most appropriate and reliable parameter.

Increased serum OPN level was reported in patients with fulminant hepatic failure and might play an important role in liver regeneration due to activation of hepatic stem cells [33]. OPN is dramatically upregulated both at mRNA and protein levels during the pathogenesis of hepatic fibrosis and in other inflammatory processes [34]. Activated hepatic stellate cells are mainly responsible for the increased expression and synthesis of OPN during fibrogenesis and the increased OPN induces collagen type I production [35]. It was observed that recombinant OPN induces type I collagen synthesis in a dose-dependent manner in cultured hepatic stellate cells [35]. Activated hepatic stellate cells also express αvβ3 integrin, the ligand for OPN [36] and the engagement of OPN with αvβ3 integrin triggers collagen synthesis [13]. Furthermore, a correlation was observed between elevated collagen-I and cleaved OPN protein in HCV cirrhotic patients compared to healthy individuals [13]. In the present study, we have observed a highly significant positive correlation between the staining intensity of α-SMA in liver biopsy specimens and serum OPN levels in HCV infected patients. Furthermore, the staining intensity of mature collagen fibers also positively correlated with serum OPN levels indicating the mutual relationship between the increased OPN expression and collagen synthesis during the progression of hepatic fibrosis in chronic HCV infection.

Since chronic HCV infection is associated with inflammation and fibrosis of the liver, a non-invasive biomarker is important to grade the disease stage and to begin therapy in order to prevent progression to HCC. Even though, increased levels of serum OPN have been reported recently, so far there was no conclusive study to use OPN as a definitive biomarker to grade hepatic fibrosis in HCV-infected patients. It was reported that increased plasma OPN concentration correlates with the severity of hepatic fibrosis and inflammation in HCV infected patients [37]. It was also observed that plasma OPN level is a potential diagnostic marker in HCV-related HCC, especially among high-risk group of patients and possesses good prognostic value [38]. Furthermore, circulating OPN level is characterized as an excellent predictor of cirrhosis in patients with hepatitis B infection [39]. Overall, it was observed that, OPN level in liver, adipose tissue and serum is correlated with fibrosis in patients with alcoholic liver disease and as a new relevant biomarker for liver fibrosis [15]. In the present study, we proved that serum OPN concentration positively and significantly correlates with the stage of fibrosis in patients with HCV infection and could be used as a biomarker as well as a prognostic index towards HCC. Since OPN is highly expressed in HCC and correlated with dismal prognosis, it is suggested that OPN enhances tumor development and metastasis [40].

In summary, the current study demonstrated that serum OPN concentrations positively and significantly correlate with the degree of hepatic fibrosis in patients with chronic HCV infection. The study also proved that serum OPN levels reflect the extent of hepatic fibrosis and could be used as a non-invasive biomarker to assess the grade of fibrosis in HCV patients that would help to reduce the number of liver biopsies. Comparative analysis of four serum biomarkers demonstrated that serum OPN is an independent predictor of the degree of hepatic fibrosis and also a prognostic index towards progression of fibrosis to HCC. However, serum OPN could be used only as an indicator for the stage of fibrosis in patients with HCV infection and further examination employing other methods is required to confirm the stage of fibrosis.
